# Thymic and Postthymic Regulation of Naïve CD4^+^ T-Cell Lineage Fates in Humans and Mice Models

**DOI:** 10.1155/2016/9523628

**Published:** 2016-05-30

**Authors:** José E. Belizário, Wesley Brandão, Cristiano Rossato, Jean Pierre Peron

**Affiliations:** ^1^Department of Pharmacology, Institute of Biomedical Sciences, University of São Paulo, 05508-900 São Paulo, SP, Brazil; ^2^Department of Immunology, Institute of Biomedical Sciences, University of São Paulo, 05508-900 São Paulo, SP, Brazil

## Abstract

Our understanding of how thymocytes differentiate into many subtypes has been increased progressively in its complexity. At early life, the thymus provides a suitable microenvironment with specific combination of stromal cells, growth factors, cytokines, and chemokines to induce the bone marrow lymphoid progenitor T-cell precursors into single-positive CD4^+^ and CD8^+^ T effectors and CD4^+^CD25^+^ T-regulatory cells (Tregs). At postthymic compartments, the CD4^+^ T-cells acquire distinct phenotypes which include the classical T-helper 1 (Th1), T-helper 2 (Th2), T-helper 9 (Th9), T-helper 17 (Th17), follicular helper T-cell (Tfh), and induced T-regulatory cells (iTregs), such as the regulatory type 1 cells (Tr1) and transforming growth factor-*β*- (TGF-*β*-) producing CD4^+^ T-cells (Th3). Tregs represent only a small fraction, 5–10% in mice and 1-2% in humans, of the overall CD4^+^ T-cells in lymphoid tissues but are essential for immunoregulatory circuits mediating the inhibition and expansion of all lineages of T-cells. In this paper, we first provide an overview of the major cell-intrinsic developmental programs that regulate T-cell lineage fates in thymus and periphery. Next, we introduce the SV40 immortomouse as a relevant mice model for implementation of new approaches to investigate thymus organogenesis, CD4 and CD8 development, and thymus cells tumorogenesis.

## 1. Introduction

In the last decades, we have much learned about basic fundaments for understanding the molecular mechanisms by which immature bone marrow CD3^−^CD4^−^CD8^−^ cells rearrange their T-cell receptors in the thymus and are successively programmed to become single-positive CD4^+^ T-, CD8^+^ T-, and CD4^+^CD25^+^ T-regulatory cells (Tregs) [1]. During a pathological condition in peripheral lymphoid organs, these T-cells become functionally and phenotypically heterogeneous populations [2]. For instance, upon antigenic stimulation by antigen-presenting cells (APCs), naïve CD4^+^ T-cells (Th0) expand and differentiate into at least five effector cell subsets referred to as Th1, Th2, Th17, IL-9-producing CD4^+^ T-cells (Th9), and T follicular helper (Tfh) cells and two regulatory/suppressive subsets referred to as induced regulatory (iTreg) T-cells named IL-10-producing CD4^+^ T-cells (Tr1) and transforming growing factor-*β*- (TGF-*β*-) producing CD4^+^ T-cells (Th3). In addition, naïve CD4^+^ T-cells (TN) will progress through central memory (TCM) T-cells and then to effector memory (TEM) T-cells and finally to terminally differentiated effector memory (TEMRA) T-cells. Expression of surface markers has been used to identify human T-cells in these various states, including CD45RA, CD45RO, CCR7, CD62L, CD27, CD28, and CD44. The CD8^+^ T-cell population also progress to central memory T-cells and finally terminally differentiated effector memory (TEMRA) T-cells [3]. All these T-cell populations are located in the follicular regions of lymph nodes and spleen and will act in coordinating the various aspects of immune response. Several advances have recently been made in understanding the signaling pathways that lead to these differentiation programs, in particular the cross-regulated feedback loops of cytokines and multiple transcription factors that facilitate a balanced immune response to pathogens or infected cells while avoiding chronic inflammation and autoimmunity. At the first part of this paper, we will provide an update on the complex intracellular signaling pathways and transcriptional factors controlling the CD4^+^ T-cell differentiation processes in thymus and peripheral lymphoid tissues. At the second part, we will introduce the SV40 immortomouse as a mouse model for new approaches and methods to examine differentiation and maturation of T-cells in the thymus and strategies to generate thymic progenitor epithelial cells for therapeutic applications.

## 2. Thymic Positive and Negative TCR-Based Selection of Immature T-Cells

**Figure 1:**
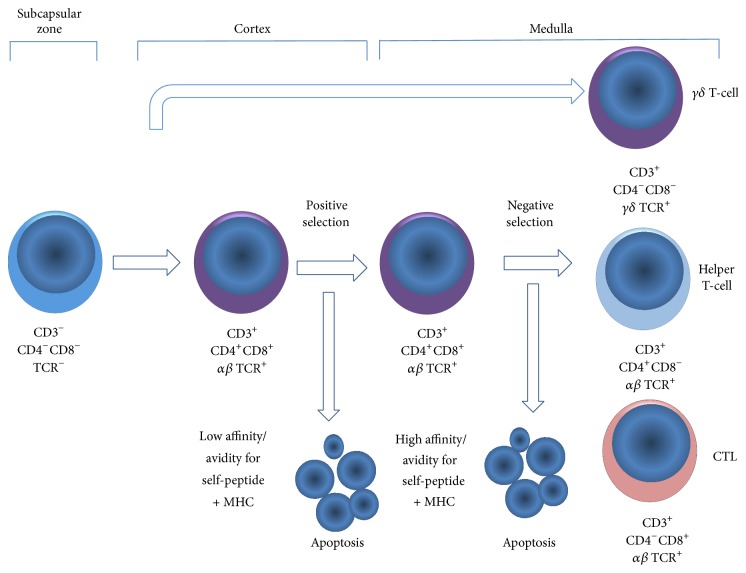
Schematic representation of T-cell positive and negative selection along the differentiation and maturation of T-cell progenitors in the thymus. Expression and rearrangement of the T-cell receptor (TCR) genes and upregulation of CD4 and CD8 give rise to CD4^+^CD8^+^ double-positive (DP) thymocytes whose T-cell receptor binds to self-antigens presented by cortical thymic epithelial cells (cTECs). Insufficient affinity for self-MHC blocks intracellular signals for cell survival and leads to cell death and positive selection at the cortex. These cells migrate to the medulla, where they bind to tissue-restricted antigens (TRA) presented by medullary TECs (mTECs). Excessive affinity for self-peptides in the context of MHC will determine cell death of autoreactive T-cells and negative selection. Only a small fraction of T-cells survive and are exported to the periphery.

The thymus, a primary lymphoid organ, is crucially necessary for T-cell development and immune responses [4, 5]. Through the actions of many hormones, cytokines, and stroma factors, a naïve thymocyte progresses into several phenotypically distinct stages, defined as double negative (DN), double positive (DP), and single positive (SP), which are characterized by the expression of the coreceptors or cluster of differentiation (CD) named CD3, CD4, and CD8 on T-cell surface membrane. The DN subset is further subdivided into four stages (DN1, DN2, DN3, and DN4/pre-DP) by differential expression of the coreceptors CD44 and CD25 [6, 7]. In addition, T-cell development is characterized by expression and rearrangement of the T-cell receptor (TCR) genes coding for *γ*, *δ*, *α*, and *β* chains. This stochastic process leads to V(D)J somatic recombination of TCR genes to give rise to either *γ* and *δ* or *α* and *β* progenitors at the CD4 and CD8 double-negative (DN) stage. This process is analogous to immunoglobulin recombination in B-cells that occurs in the bone marrow. TCR*γ* and TCR*δ* chains are expressed by only 2–14% of peripheral T-lymphocytes. T-cells bind to intrathymic antigen peptides presented by major histocompatibility complex (MHC) class I and II molecules on the surface of dendritic cells (DCs) and thymic epithelial cells (TECs). The positive selection of CD4^+^ T-cells depends on class I expression whereas that of CD8^+^T depends on class II expression in cortical epithelial cells. Thus, if TCRs on T-cell membrane recognize with high affinity self-antigens using class I MHC molecules, the cell eliminates CD4 expression and remains TCR^+^CD3^+^CD8^+^. If its TCRs recognize self-antigen using class II MHC, the cell eliminates CD8 expression and remains TCR^+^CD3^+^CD4^+^ (Figure 1). The positive selection rescues from apoptotic cell death all thymocytes capable of self-peptide MHC recognition [8]. Next, the positively selected cell population undergoes negative selection that kills by apoptosis all thymocytes identified by their ability to recognize self-peptide presented in the context of MHC I and MHC II complexes, for example, autoreactive cell clones. Among the molecules implicated in T-cell apoptosis are Nur77 protein, a member of the orphan nuclear receptor superfamily, and the Bim protein, a Bcl-2 family member [8]. There are various mechanisms operating in these events to ensure tolerance to self, including clonal deletion, clonal diversion, receptor editing, and anergy [7]. Negative selection saves self-reactive clones with suppressive or regulatory activity based on self-reactive TCRs to self-peptides, the expression of CD25 differentiation antigen, and the associated transcription factor forkhead box P3 (Foxp3) [9]. This mechanism is essential for the establishment of central and peripheral T-cell tolerance [7]. At the end, a relatively small number (fewer than 5%) survive from positive and negative selection in the thymus and will constitute the mature CD4^+^ and CD8^+^ population into periphery pool [5].

A number of the transcription factors including the Th-POK (T-helper-inducing POZ/Kruppel-like factor), GATA3 (GATA-binding protein 3), and RUNXs (Runt-related transcription factor) are required for intrathymic differentiation of T-cells precursors into specialized T-cell clones [10–12]. CD4^+^ T-cells are MHC II restricted and exert helper functions, whereas CD8^+^ T-cells are MHC I restricted and exert cytotoxic functions. The Th-POK gene is upregulated in MHC II restricted thymocytes as they undergo CD4-lineage differentiation. In contrast, MHC I restricted cells upregulate Runx3 gene, as they undergo CD8-lineage differentiation [11, 12]. In fact, some reports have also demonstrated that both Th-POK and RUNX3 transcription factors are required for the differentiation of a population of intraepithelial lymphocytes (IELs) known as CD4^+^CD8*αα* ^+^ into the periphery pool.

## 3. Mosmann and Coffman's Polarization Signaling Model for CD4^+^ T-Cell Lineage Differentiation

In 1989, Mosmann and Coffman [13] proposed the original Th1-Th2 paradigm to explain a natural tendency of an immune response to become progressively polarized and finally acquire an effector function. In both murine and human immune systems, the Th1-to-Th2 differentiation process requires distinct production of the autocrine growth factors, cytokines, and their receptors that will exert suppressive activities on each other's development or antagonism [14, 15]. Recent studies have shown that the populations of effectors and memory lymphocytes formed in this process are extremely heterogeneous in terms of phenotype, function, and longevity [16, 17]. A naive CD4^+^ lymphocyte acquires an effector function as T-helper cell 1 (Th1), Th2, Th9, Th17, Tfh, and antigen-specific regulatory cells depending on certain critical integrated signals derived from TCRs, costimulatory molecules, and intense cytokine cross talk [16–20]. The experimental observations from many studies showing phenotypic diversity have been applied for the development of a mathematical modeling approach to study the CD4^+^ T-cell differentiation, plasticity, and heterogeneity [21]. Next, we will shortly summarize current knowledge on important signaling pathways and mechanisms for the commitment of specific T-cell lineages.

## 4. Th1

**Figure 2:**
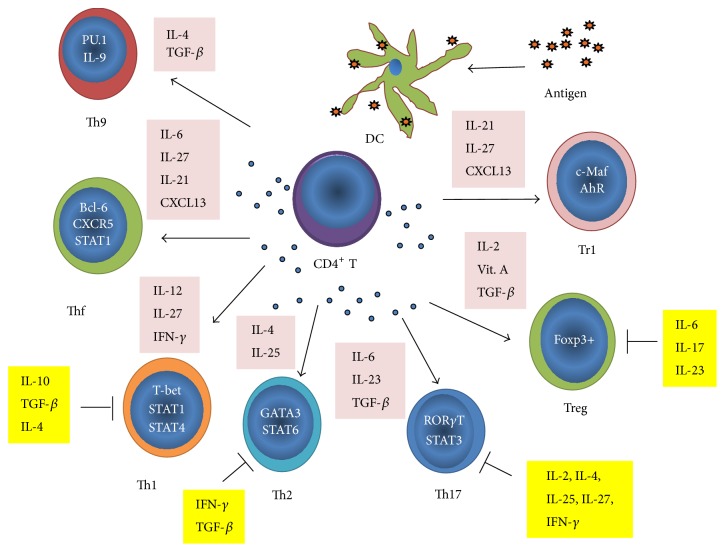
Schematic representation of the cytokines and transcription factors controlling CD4^+^ T-cell differentiation. Upon antigenic stimulation by antigen-presenting cells, naïve CD4^+^ T-cells (Tho) expand and differentiate into at least seven effector cell subsets. Each of these phenotypes is induced by a signature pattern of cytokines (pink box) and multiple transcription factors (blue, nucleus) and regulated by distinct cytokines (yellow box). Some cytokines promote the clonal expansion of naïve antigen-specific T-cells and the acquisition of T-cell effector functions whereas others such as TGF-*β*, IL-6, IL-9, IL-17, IL-22, and IL-23 avoid redifferentiation and conversion of Treg cells into Th1/Th17 pathogenic phenotype. The phenotypic plasticity that drives conversion of some Th-subtypes to another Th-type is a mechanism not yet fully understood.

Naïve CD4 T-helper cell-derived subsets are characterized on the basis of the expression of one or more master specific transcription factors and production and/or response to specific set of cytokines (Figure 2). The transcription factors T-bet and STAT1 (signal transducers and activators of transcription family of transcription factor 1) are master regulators for Th1 cell differentiation and expansion [18–20]. The Th1 cells are capable of producing IL-2, IL-18, IFN-*γ*, and TNF-*β* (lymphotoxin). Th1 cells can mediate macrophage activation and delayed type hypersensitivity, which are collectively termed cell-mediated immune responses. IFN-*γ*, TNF-*α*, IL-2, and lymphotoxin-*β* activate macrophages and CTLs which kill intracellular (type 1) pathogens, such as* Listeria monocytogenes* and* Leishmania*.

## 5. Th2

Th2 helper cell subset is characterized by the presence of the master specific transcription factors GATA3 (GATA-binding protein 3) and STAT6. IL-4 produced by activated macrophages and DCs is the most important cytokine for induction of these transcription factors and Th2 differentiation. Th2 cells promote secretion of IgG1 and IgE by B-cells and promote immediate-type hypersensitivity reactions, collectively termed humoral immunity. The Th2 response leads to antibody production and is associated with chronic infections, such as retroviral infections and various extracellular (type 2) pathogens, including helminthes and nematodes. The production of cytokines, including IL-4, IL-5, IL-10, IL-13, IL-25, IL-31, and IL-33, by mast cells, eosinophils, and NKT cells, shifts the balance from a Th1 to a Th2 response, thereby leading to the activation of B-cell clones containing the repertoires of antibodies for a specific immune response, including the production of IgG1 and IgE [18, 19]. The Th2 response produces the suppressive cytokines IL-10 and TGF-*β*, which dampen the protective Th1 response [22]. Thus, Th1 cells have been considered to be responsible for some organ-specific autoimmune disorders, whereas Th2 cells have been shown to be critical for the development of allergic inflammation [23].

## 6. Th17

Th17 helper cell subset is characterized by the presence of the master transcription factors ROR*γ*t (retinoic acid receptor-related orphan receptor gamma t) and STAT3 [24, 25]. Th17 cells development from naïve T-cells is promoted by IL-6 and TGF-*β* [24, 25], whereas early differentiation of Th17 cells is suppressed by IFN-*γ* and IL-4 [23, 26, 27]. However, committed (mature) Th17 cells are resistant to IFN-*γ* and IL-4 suppression, and, likewise, mature Th1 and Th2 cells are resistant to IL-4 and IFN-*γ* mediated suppression, respectively [26, 27]. Vitamin A obtained from the diet is converted into retinoic acid (RA) by CD11c^+^CD103^+^ lamina propria dendritic cells [28]. RA is capable of inhibiting the TGF-*β*- and IL-6-driven induction of Th17 [29]. Th17 cells are abundant in intestinal lamina propria cells and mesenteric lymph nodes, where they have an important role in the clearance of a variety of commensal bacteria [16, 30, 31]. The cytokines IL-6 and TGF-*β*, at low concentrations, induce Th17 differentiation (differentiation phase) [25]. Next, Th17 cells initiate the production of a large range of cytokines, including IL-17A, IL-17A/F, IL-10, IL-21, and IL-22, which contribute to the amplification phase of Th17 differentiation [32]. IL-17 molecules secreted by Th17 cells activate stromal cells, endothelial cells, and other cells to produce more proinflammatory mediators, including IL-1*β*, IL-6, IL-8, TNF-*α*, GM-CSF (granulocyte-macrophage colony stimulating factor), NO (nitric oxide), and metalloproteinases [27, 33].

The results of many studies have supported the pathogenic role of Th17 in the course of various human inflammatory diseases, including experimental autoimmune encephalomyelitis (EAE), multiple sclerosis, collagen-induced arthritis, and inflammatory bowel disease in murine models [23]. Th17 cells become pathogenic at later steps when their production of cytokine IL-23 increases, promoting the stabilization phase of Th17 differentiation [30, 34]. Nonetheless, there have been many debates questioning the pathogenic role of Th17 cells in human patients with autoimmune diseases mainly because of the results of studies that compare mouse Th17 cells to human Th1 cells producing IFN-*γ* or IL-12 [16, 23, 32, 34–37]. The most effective cytokines to enhance the generation or expansion of human Th17 cells are IL-1*β* and IL-23, whereas IFN-*γ*, IL-4, and IL-27 suppress their generation [37, 38]. Overall, these studies have reinforced the notion that the longevity, stability, and plasticity of Th17 cells can be influenced by the variations in the cytokine milieu as well as the presence of stromal cells and Tregs cells [16, 20, 23, 35, 37]. In a recent study, Gagliani and colleagues using different transgenic mice models elegantly showed that Th17 transdifferentiate into regulatory T-cells and contribute to the resolution of inflammation [39]. Therefore, the development of new biological approaches and animal models to evaluate key regulatory drivers in the Th17 cell reprogramming will help in understanding their role at physiological conditions and in various immune-linked diseases.

## 7. Th9

Th9 or IL-9-producing CD4^+^ T-cell is a discrete T-helper subset that develops from naïve T-cells in the presence of TGF-*β* and IL-4 [40–42]. This new subset of the T-helper population is characterized by their ability to produce large quantities of IL-9. Their differentiation requires the expression of transcription factors STAT6 (signal transducer and activator of transcription 6), PU.1, IRF4 (interferon response factor 4), and GATA3 [40, 42]. Th9 cells are activated by epithelial cell-derived cytokines, including IL-25 and IL-33, which have been implicated in the initiation of asthma. Th9 acts as a major contributor to the onset and progression of many types of allergies induced mouse models [42, 43]. This was confirmed using IL-9-producing reporter mice. Licona-Limón and colleagues showed that Th9 is essential to combat intestinal worm* Nippostrongylus brasiliensis* [44]. Interestingly, studies have also demonstrated that Th9 cells have a protective role against tumor growth [43].

## 8. Tfh

CD4^+^ T-lymphocytes can also differentiate into specialized effector follicular helper (Tfh) T-cells which display high levels of the surface receptors ICOS (inducible T-cell costimulator), CD40L (CD40 ligand), CD134/OX40, PD-1 (programmed death ligand-1), BTLA (B- and T-lymphocyte attenuator), and CD84, the cytokine IL-21, SAP (the cytoplasmic adaptor protein SLAM-associated protein), and the Bcl-6 (transcription factors B-cell lymphoma 6) and c-Maf (avian musculoaponeurotic fibrosarcoma oncogene) [45, 46]. Tfh cells induce the development and antibody isotype switching in germinal centers (GCs) into lymph nodes where final B-cell maturation occurs. T- and B-cell interactions increase Bcl-6 expression in activated CD4^+^ T-cells. This in turn increases the expression of the IL-6 receptor, IL-21 receptor, and CXCR5 (receptor for the chemokine CXCL13), which are the specific molecular markers of Tfh cells. CXCR5 ligand CXCL13 drives T-cells to B-cell border and interfollicular zones within GCs. Bcl-6 and c-Maf may synergize to generate Tfh cells by regulating the expression of critical factors such as IL-21, IL-21 receptor, and CXCR5 [46]. Similar to Th17 cells, the ability of IL-6 to promote Tfh cell differentiation depends on the action of IL-21. The expression of Blimp-1 (B-lymphocyte-induced maturation protein-1) may help to maintain tolerance by inhibiting Bcl-6 expression and further differentiation of Tfh cells [47]. The costimulatory receptor ICOS signaling is very important for the differentiation of Tfh cells since it induces the transcription factor c-Maf, which in turn induces IL-21 expression. Finally, the natural follicular Treg cells exist which limit GCs reactions by suppressing the responses of both Tfh and GC cells [48].

## 9. Tregs

The regulatory CD4^+^ T-cells (Tregs) are defined by cellular surface expression of CD4^+^ CD25^+^ (IL-2R *α* chain receptor), OX40/CD134, CD27, CTLA-4 (CTL-associated protein 4), CD62-L, and TGF-*β* [1, 9, 18, 49–53]. This natural Treg subset (nTreg) develops continuously after 3-4 days after birth in the mouse thymus from CD4^+^ CD8^−^CD25^+^ or CD4^+^ CD8^−^ CD25^−^ thymocytes upon initiation of the expression of the forkhead family transcription factor Foxp3 (forkhead box P3) that is upregulated following stimulation by thymic stromal derived lymphopoietin [19, 54]. Removal of the thymus from 3-day-old neonatal mice leads to multiple organ autoimmune disease in some strains of mice [54]. Besides nTreg, a peripheral induced Treg (iTregs) population arises after antigen priming under certain cytokine milieu in peripheral lymphoid organs [18, 33, 50, 55]. The generation of iTregs occurs under the environment rich in TGF-*β* and retinoic acid [29, 56]. Retinoid acid can shut down the synthesis of cytokines IFN-*γ*, IL-4, and IL-21, which in concert promote an inhibitory effect on Foxp3 mRNA and protein induction [29, 56]. The naturally arising Treg cells exert suppressor and regulatory functions that are vital to maintain the delicate balance between tolerance and protective immunity. Tregs control autoimmune responses but also limit the onset of effective antitumor immune responses. Tregs cells comprise about 5–10% of the mature CD4 helper T-cell subpopulation in mice and 1-2% of CD4^+^ helper T-cells in humans [18], but cellular and molecular mechanisms of human Tregs remain incompletely characterized [9, 19, 57]. It seems that the same antigenic peptide can stimulate both CD4^+^ helper and regulatory T-cells depending on peptide affinity, expression level, and cytokine milieu.

The expression of Foxp3 is required for the expression of GITR (the glucocorticoid-inducible tumor necrosis receptor), CTLA-4, KLRG1 (killer-cell lectin-like receptor G1), CD25, and Blimp-1 (B-lymphocyte-induced maturation protein-1) which are involved in the regulatory cell development by controlling a genetic program that specifies this cell lineage and by repressing alternative cell fates [18, 50–53]. Over 300 proteins are necessary to ensure the high expression of Foxp3 protein and lineage stability of Tregs [1]. Foxp3-induced inhibition of the gene for IL-2 and upregulation of the gene for CD25 are due to its binding and repression to the NF-*κ*B, AML1/Runx1, and NFAT transcription factors [18, 51]. Generally, Tregs need to be activated to exert their suppressive function due to their natural anergic state. Prior to activation, the binding of IL-2, via IL-2 receptor CD25, and also IL-4, IL-7, and IL-15 is responsible for survival and maintenance of Tregs, which divide continuously and acquire an activated/memory phenotype.

Deficiency of Tregs causes autoimmune diseases and predisposes to solid organ and hematopoietic stem cell graft rejection. Several putative mechanisms by which Tregs promote the suppression of effector T-cell response have been investigated [9, 19, 55, 57]. These include the Treg cell contact inhibition of APCs and activated T-cells, Treg-induced killing of either APCs or T-cells or both [18, 58], and Treg-induced suppression of activated T-cell via expression of cytokines IL-10 [15] and TGF-*β* [22, 59, 60] and overexpression of PD-L1 and generation of DCs with reduced capacity to stimulate effector T-cell responses [57]. TGF-*β* contributes, at least in part, to the* in vivo* suppression of effector T-cell response. Latent TGF-*β* and a latency-associated peptide (LAP) are constitutively present on the surface of Tregs [22]. The activation occurs after TGF-*β* cleavage by plasmin or other alternative mechanisms [22]. TGF-*β* signals through the T*β*RI and T*β*RII (type I and type II TGF-*β* serine-threonine kinase receptors), which is followed by phosphorylation of Smad 2/3 and Smad 4. Smad 7 and Smad ubiquitin regulatory factors (Smurfs) exert negative feedback by inducing T*β*RI and T*β*RII degradation, thereby stopping the binding of TGF-*β* into its plasma membrane receptor [22, 60].

Many different* in vivo* and* in vitro* assays have been used to investigate the mechanisms by which Tregs mediated suppression of immune response [53, 55, 61]. They are evaluated first by the ability to suppress conventional T-cell proliferation at 1 : 2 to 1 : 4 Treg to conventional T-cell ratios in the “classical”* in vitro* suppression assay. The granzyme-dependent and perforin-dependent mechanism is the main assay used to examine Tregs mediated killing of responder T-cells [62]. Investigations in this pathway led to the discovery that Tregs release nucleotide adenosine, a negative signal to responder T-cells via upregulating intracellular cyclic AMP, thereby reducing IL-2 production that causes inhibition of T-cell proliferation. The pericellular adenosine is catalyzed by CD39 (ectonucleoside triphosphate diphosphohydrolase 1) and CD73 (ecto-5′-nucleotidase) which are preferentially expressed by Tregs [63, 64]. Finally, Tregs stimulate DCs to express the enzymes indoleamine 2,3-dioxygenase (IDO1) and tryptophan 2,3-dioxygenase (TDO2). IDO1 is the rate-limiting enzyme controlling the degradation of the essential amino acid tryptophan into a series of metabolites named kynurenines [65]. The exact cellular pathway by which IDO leads to Treg differentiation is debatable [66]. Recently, some studies have pointed to a strong role for the aryl hydrocarbon receptor (AhR), a ligand-operated transcription factor, in T-cell differentiation [67]. The activation of the AhR by 2,3,7,8-tetrachlorodibenzo-p-dioxin (TCDD, dioxin), a potent environmental toxicant, will lead to the differentiation of naïve CD4^+^ T-cells into Foxp3 regulatory T-cells. Thereafter, several reports described that L-kynurenine acts as natural AhR ligand because of its capacity to phenotypically alter DCs. Both of these APC modifying pathways appear to be dependent on CTLA-4 (CD152), CD80, and CD86. T-cells expressing CTLA-4 can downmodulate CD80 and CD86 expression, thereby impairing the function of DCs [55].

Tr1 is another Treg cell lineage that is induced by antigen stimulation via an IL-10-dependent process in the periphery [68, 69]. Tr1 secretion of the high levels of immunosuppressive cytokine IL-10 and medium levels of TGF-*β* is required to dampen autoimmunity and tissue inflammation. Tr1 is characterized by the expression of the transcription factor c-Maf, AhR, and the costimulatory receptor ICOS. c-Maf activation leads to enhanced production of IL-21 whereas ICOS promotes the expression of IL-27. IL-21 drives the Tr1 clone expansion whereas IL-27 drives the Tr1 differentiation. In contrast, granzyme B and IL-10 expression mediate the contact-dependent suppressive activity of Tr1 cells [70]. Conventional DCs treated with immunomodulatory cytokines such as IL-10, TGF-*β*, IFN-*α*, or TNF-*α* are converted to tolerogenic type and induce the differentiation of Tr1 [71, 72]. In addition, the immunosuppressive drugs vitamin D3 and dexamethasone are also able to induce the development of Tr1 cells [72].

The transforming growing factor-*β*- (TGF-*β*-) producing CD4^+^ T (Th3) is a distinct Treg cell lineage that exerts suppressive/regulatory activities that were originally identified in mice after oral tolerance induction to myelin basic protein [73]. They produce TGF-*β* together with various amounts of IL-4 and IL-10. These cytokines influence the functional activity of multiple cell types that probably have a major role in many aspects of immune regulation and T-cell homeostasis [72].

A small subset of CD8^+^CD25^+^ T-cells sharing similar characteristics with CD4^+^ CD25^+^ Treg have been detected in human fetal and postnatal thymuses [74]. Both CD4^+^CD25^+^ and CD8^+^ CD25^+^ human thymocytes express Foxp3 transcription factor and cell surface molecules GITR, CD103, CCR8, and TNFR2 and cytoplasmic CTLA-4 proteins, which are common features of mature Treg cells. Following activation, they do not proliferate or produce cytokines but express surface CTLA-4 and TGF-*β*1. CD8^+^ CD25^+^ Tregs suppress the proliferation of autologous CD4^+^ CD25^−^ thymocytes to allogeneic stimulation by a contact-dependent mechanism related to the combined action of surface CTLA-4 and TGF-beta leading to the inhibition of the IL-2R alpha chain expression on target T-cells. Lastly, both CD4^+^ CD25^+^ and CD8^+^ CD25^+^ Treg thymocytes exert strong suppressive activity on Th1, but much lower on Th2 cells, since these latter may escape from suppression via their ability to respond to growth factors other than IL-2.

It has been demonstrated that CD8^+^ Tregs are induced in humans after different immunotherapy regimen in various pathophysiological situations. These CD8^+^Foxp3 Treg cells are able to suppress CD8^+^ responses far more effectively than naive CD4^+^Foxp3 Treg [75, 76]. Conventional dendritic cells (cDCs) and plasmacytoid DCs appear to play a critical role in the induction of CD8^+^ Tregs in bone marrow allogeneic transplantation and consequently graft-versus-host disease (GVHD) in humans, mice, and rats [76]. In organ transplantation, CD8^+^ Tregs were found in higher numbers in the graft and spleen* in vivo* of donor-specific blood transfusion-induced tolerance and anti-ICOS-treated mice [76].

Some mutations of Foxp3 gene, an X chromosome-encoded gene mapped to chromosome Xp11.23-Xq13.3, cause a rare X-linked fatal autoimmune disease in humans named IPEX syndrome which is associated with immune dysregulation, polyendocrinopathy, enteropathy, and X-linked gene [77, 78]. The fatal outcome of this autoimmune disease is a cytokines storm including the release of TNF-*α* and IL-2 cytokines in high amounts and consequently a massive and fatal aggressive myelo- and lymphoid proliferative syndrome. The* scurfy* is a mouse mutant strain that displays an analogous phenotype of human lymphoproliferative syndrome caused by a similar mutation in the ortholog mouse Foxp3 gene [79, 80]. Along the disease, mice present impaired development of suppressive Treg cells which leads to diabetes mellitus, exfoliative dermatitis, thyroiditis, enteropathy, and inflammatory bowel disease [78, 79]. Transgenic mice models bearing complete knockdown or conditional loss-of-function mutation in the Foxp3 gene die at first weeks of age due to lethal autoimmune syndrome [50]. The analyses of lymphoid and myeloid tissues revealed massive proliferation of T-cells specific for self- and environmental antigens as well as huge accumulation of dendritic cells and granulocytes in the tissues of transgenic animals [50].

Treg cellular therapy is an attractive new therapy for autoimmune diseases and transplantation and many clinical trials to validate Treg therapy in humans have been tested [81]. However, it is not yet technically possible to generate clinical grade self-Ag specific Tregs and different technological issues must be first evaluated. Treg manufacturing technology is only capable of large-scale production of polyclonal antigen-experienced effector Tregs. Different mechanisms operate for thymic and peripheral Tregs development. The mTECs are the most important thymic antigen-presenting cells for Treg cell selection in the thymus [9]. Therefore, the development and use of new mouse strain need to be explored as experimental model that will allow direct assessment of factors that drive Treg homeostasis, phenotypic conversion, expansion, survival, and functional maturation, and evaluation of proof-of-principle experiments for Treg-based therapies.

## 10. Transgenic Mice Models for Investigating Thymic Functions

Various transgenic mice models have been created for expression or deletion of critical molecules and pathways of signal transduction of CD4^−^CD8 lineage differentiation, negative selection to tissue-specific antigens, T-cell-mediated autoimmunity through central tolerance, and Treg cell generation [82, 83]. The development of genetic engineering strategies for creation of immunodeficient and chimaeras mice models that successfully engrafted and developed human myeloid and hematopoietic cells into host organs and tissues has provided helpful assay system to model thymic processes [82–85]. The NOD/scid/IL2rg^null^ mouse strain named NSG mice, which lacks T-, B-, and NK cells, is the only one that allows more complete human immune cell system reconstitution as compared with other immune deficient mice [86]. On the other hand, the oncogenic retroviruses- and murine polyomavirus-induced transgenic models have proven extremely successful to investigate host cells and viral protein interactions in different tissues and cells [87].

The thymus is a pharyngeal organ formed by ectoderm and endoderm layer cells derived from the neural crest that is located in the chest directly behind your sternum and between your lungs. The primary immunological function of the thymus is the production of self-restricted and self-tolerant T-cells. At embryonic stage E11.5, primordial TEC progenitor cells meet hematopoietic cells through discrete thymic microenvironment in the thymic anlage [4, 6, 88]. The interaction and network of mesenchyme, epithelium, and thymocytes result in early differentiation of epithelial cells and the lymphostromal stroma. Wnt glycoproteins of the int/Wingless family bind to complexes of cell membrane frizzled (fz) receptors leading to activation of *β*-catenin, which translocate to the nucleus where *β*-catenin associates with T-cell factor (TCF), a DNA-binding protein family, and activate TCF and lymphoid enhanced factor (LEF) transcriptional activities. The transcription factors autoimmune regulator (AIRE) [89–91] and forkhead box N1 (FOXN1) [92, 93] are the most important transcription factors for thymic organogenesis and TECs differentiation. Expressed exclusively in thymic and cutaneous epithelia, they are required for cortical (c) and medullary (m) TECs differentiation and the establishment of thymic stroma. Although the cortical and medullary TECs of normal mouse express high levels of MHC II molecules, there are substantial differences in the antigen-presenting pathways by these cells [94].

Mice with deletion of AIRE gene developed Sjögren's syndrome, a severe autoimmune disease driven by defective negative selection that results in the presence of autoantibodies to *α*-foldrin in multiorgans [8, 82, 89, 90]. AIRE regulates the antigen presentation by mTEC and medullary thymic dendritic cells during negative selection [95]. In human, AIRE mutation is responsible for autoimmune polyendocrinopathy candidiasis ectodermal dystrophy (APECED) [96].

FOXN1 is not required for thymus organogenesis but is required for development of thymic epithelial cells. However, in the absence of functional FOXN1, TECs are arrested at an immature progenitor stage because of the absence of thymic epithelial progenitor cells [97]. The athymic* Nude* mice contain a mutation at FOXN1 and do not develop intrathymic T-cells but develop extrathymic T-cell differentiation [93, 98]. Mutations in FOXN1 gene in human cause alopecia and nail dystrophy [99]. The DiGeorge Syndrome (DGS) is the human prototype for severe defects in T-cell differentiation due to thymic hypoplasia. This syndrome may arise from mutations affecting the transcription factor TBX1 (T box 1) that controls whether cells can become TECs.

The thymus undergoes a dramatic age dependent involution due to loss of epithelial-mesenchymal interactions with TECs, dendritic cells, vasculature, and mesenchymal cells that provide signals for their survival, proliferation, and differentiation [100, 101]. The thymus produces all of your T-cells along puberty and the peripheral T-cell expansion seems to play a greater role in protecting older people from diseases. Molecular analysis of signal-joint T-cell receptor (TCR) excision circles (sjTRECs) in recent thymic emigrants (RTEs) showed that they significantly decrease with increasing age in murine thymus tissue [102]. A mathematical model suggests that decreases in T-cell repertoire are not associated with thymic involution but rather with peripheral selection due to accumulation of mutations and consequently a decline in the TCR repertoire [103]. The rapid ageing process has been associated with reduced production of growth factors such as thymosin, the hormone of the thymus, fibroblast growth factors (FGF-7 and FGF-10), insulin-like growth factor (IGF-1), growth factor (GH), keratinocyte growth factor (KGF), leptin, and cytokines IL-7, IL-10, and IL-22 which are essential for all adult TECs expansion and differentiation [102, 104]. On the other hand, it was shown that increased mRNA expression of leukemia inhibitory factor (LIF), stem cell factor (SCF), IL-6, and M-CSF correlated with age [105]. It is interesting that androgen blockage or sex steroid ablation (SSA) induces thymic regrowth with rapid changes of genes, including genes of the Wnt pathway, which has an important role in ageing and regeneration [106].

Immune system ageing (immunosenescence) occurs with a significant decrease of germinal centers, CD8 T-cells, naïve T-cells, and IgM-expressing B-cells [105]. The development of novel pharmacological strategies for recovery of immunosenescent cells using thymopoietic factors and stem cell transplantation will have a significant impact on lifespan and global public health [102, 104]. TECs progenitor cells are characterized by two surface markers named Mts20 and Mts24 and also keratin types 5 and 8 [97]. Because of their capacity of self-renewal and differentiation potential, studies have been done to explore their capability of repopulation of the entire thymic epithelium into immunodeficient mice, but definite proof of their application in humans is still lacking [6, 97]. Investigations on alternative cellular and molecular mechanisms leading to thymic defects and severe combined immune deficiencies (SCID) in humans depend on the creation of novel mice models which have been essential for development of innovative and safe therapies.

## 11. Immortomouse

**Figure 3:**
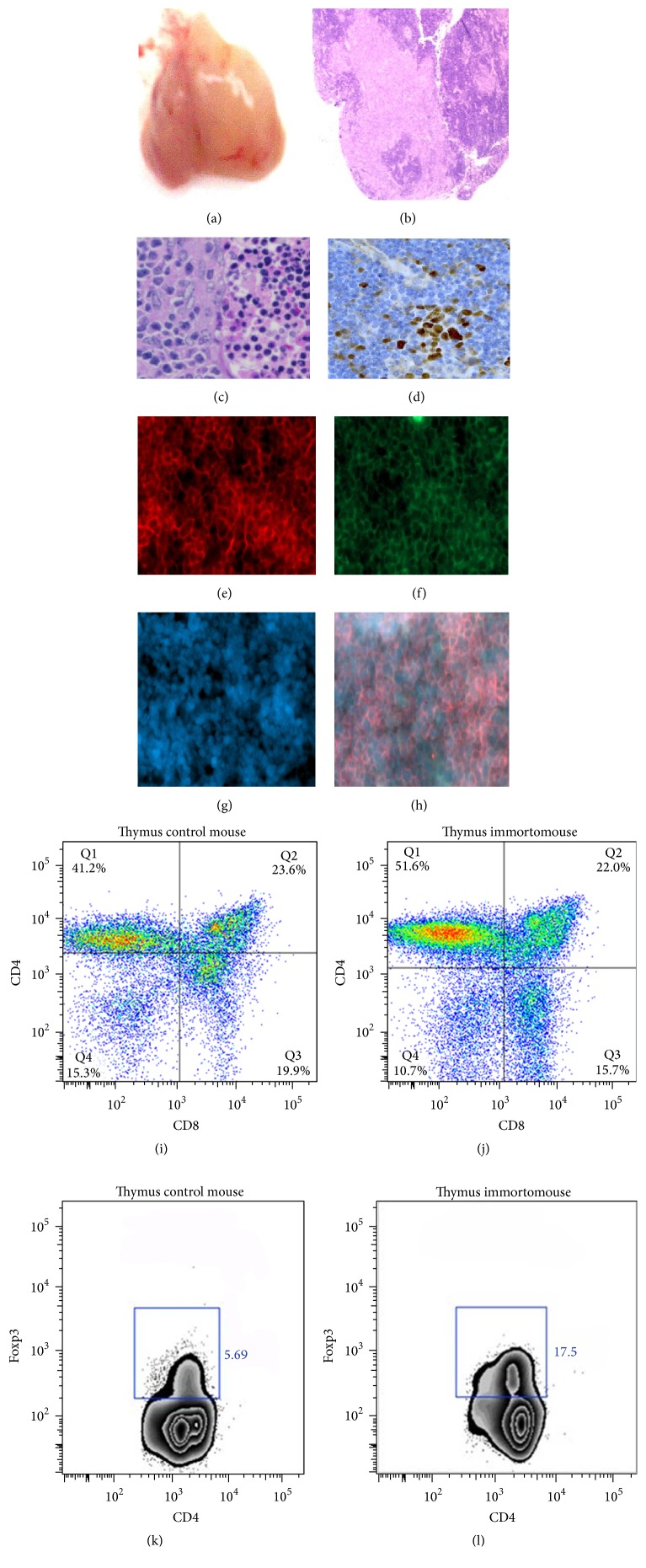
Anatomic macroscopic and microscopic structures of a large thymus ((a), (b), (c), and (d)) obtained from an immortomouse at three-month age. The panoramic view shows that the morphology and epithelial and lymphoid structures are preserved. The dense lymphoid tissue in the thymic cortex and medulla areas ((b) and (c)) indicates active germinal centers for T-cell differentiation. The tissues were stained for immunohistochemistry with hematoxylin and eosin (c) and anti-large T-SV40 monoclonal antibody (d). The phenotypic characterization of the thymus and spleen CD4 lineages by fluorescent microscopy analysis of labeled cells ((e), (f), (g), and (h)) and flow cytometric analysis ((j), (k), and (l)) indicated that pre-T-cells progressed through their development to become specific subtypes CD4^+^, CD8, and double-positive CD4^+^/CD8^+^ (j) as observed in the control mice (i). The rates of CD4/Foxp3 expression in T-cell populations expanded 9-fold in the hyperplasic thymus (l) as compared to control (k). All cells were labeled using fluorescein-conjugated anti-mouse CD3, phycoerythrin-conjugated anti-mouse CD4, and allophycocyanin- (APC-) conjugated anti-mouse CD8 mAbs and Alexa 488-conjugated anti-mouse Foxp3 mAbs (BD Biosciences). Numbers refer to percentages of cells in marker region after subtraction of other subpopulations.

Immortomouse is a transgenic mouse strain that expresses a simian virus 40 large T-antigen named tsA58 (Tag), a mutant temperature-sensitive nuclear phosphoprotein involved in viral replication [107]. The tsA58 transgene is located on chromosome 16 at 1.4 kb upstream of the chromosomal marker D16Mit30 and has a size of 6.9 kb [108]. The transgene expression is under the control of murine H-2K^b^ promoter that is responsible for inducing the expression of MHC class I antigen. Thus, the transgene is inducible by exposure to interferon-*γ* under permissive conditions of 33°C [107]. We have crossed immorto transgenic C57/BL10 hybrid mouse strain with wild BALB/c mouse strain to over 20 generations to generate a pure BALB/c immortomouse strain. The only observable phenotype in the BALB/c mouse strain is the thymus overgrowth that can weigh over two grams in both homozygous and heterozygous adult mice at six months of age [109], as it has been described in original work [107]. Both homozygous males and hemizygous females do not succumb to any pathological disease within this period. In fact, histological analysis showed that hyperplastic thymi had a relatively normal organization and displayed no characteristics of thymoma or thymic lymphoma (Figure 3). Our immunohistochemistry study revealed that SV40 antigen is expressed in the thymus tissue, mainly in T-cells (Figure 3). In the first analysis of the phenotype of immortomouse, Jat and colleagues concluded that the enlarged thymic histology and T-cell repertoire and polyclonality expansion were normal in transgenic animals. They did not observe tumor formation in syngeneic recipients but could not exclude the late transformation of highly proliferative cells [107, 110].

It is well characterized that large T-antigen tsA58 immortalizing gene expression can overcome functional and differentiation properties into target cells and stimulate cell growth without oncogenic transformation [107, 110]. The SV40 expression has been shown to freezing cells at lineage-specific development, which allows a number of advantages. Many conditionally immortalized cell lines directly derived from tissues and organs have been isolated [110, 111]. The growth of these cell lines is temperature-dependent since they grow at 33°C in the presence of IFN-*γ* but arrest at non-permissive (39°C) temperatures [111]. The SV40 large T-antigen (Tag) is a multifunctional protein that displays DNA helicase, RNA helicase, and ATPase activities [112, 113]. Among several proposed mechanisms in the literature for SV40 immortalization, the control of the cell cycle and apoptosis via interaction and rapid degradation of p53 and RB proteins is the most well established [112–114]. By inactivating RB proteins (pRb, p130, and p107), Tag removes the normal G1-phase to S-phase cell cycle checkpoint thereby facilitating uncontrolled cycling and cell growth. In 2013, Sadasivam and DeCaprio identified an 8-protein complex that specifically interacts with the retinoblastoma-related proteins p130 and p107 and named it DREAM (DP, RB-related, E2F, and MuvB) [115]. The mammalian DREAM binds to the promoters of all cell cycle regulated genes and promotes their repression during cellular quiescence [115]. Large T-antigen directly binds to DREAM associated proteins and can restore cell cycle program [116].

To our knowledge, there have been no reports to date examining the homeostatic expansion of lymphoid T-cell subtypes in the tsA58 transgenic model. The presence of CD3^+^, CD4^+^, CD8^+^, and Foxp3 lineages at early and late time of thymic hyperplasia indicated that they are functional (Figure 3). We cannot conclude whether T-cell lineages did efficiently develop T-cell antigen receptor (TCR) in the thymus* in vivo* (Figure 3). To confirm this, we performed antigenic stimulation assays using T- and B-cell populations collected from the spleen of immortomice. We observed normal T and B proliferation stimulated by Concanavalin A and lipopolysaccharide (LPS), respectively. On the contrary, T-cells underwent apoptosis when stimulated with staurosporine or glucocorticoids (data not shown). Treg cells comprise about 5–10% of the mature CD4^+^ and CD3^+^ subpopulation in the lymphoid organs. However, the rate of CD4^+^ CD25^+^ Foxp3 population expanded 9-fold in the hyperplasic thymus, as compared to population in the organs from normal wild type mice (Figure 3). These results demonstrated that Treg cells were converted to the effectors and memory cells. IFN receptors are expressed and IFN-*α* and IFN-*β* are secreted in the thymus and medulla in normal fetal and postnatal thymus in the absence of infection [117]. We hypothesized that IFN-*α*/*β* may have a direct role in stimulation of the interferon inducible murine H-2Kb promoter and consequently large T-antigen tsA58 gene expression in the thymus tissue cells of immortomice. This and other* in vitro* and* in vivo* studies are underway to delineate the molecular and cellular mechanisms by which SV40 virus large T-antigen regulates T-cell expansion and function.

A report in the literature described a transgenic mouse model expressing cyclin D1 from keratin 5 promoter (K5.CyclinD1) that resembles the immortomouse phenotype [118]. The offspring of cyclin D founder mice showed dramatic and continuous growth that resulted in thymus hyperplasia (~2 g). The thymic tissue did not undergo ageing-associated involution, and its large size caused the death of mice due to severe respiratory distress [118]. The thymocytes histology appeared to be normal and did not show evidence of transformation [118]. Nonetheless, it is known that overexpression of cyclin D1 is a common driver for uncontrolled growth regulatory pathway in cancers [119].

An increasing number of targets of large T-antigens have been identified including p53 and RB family of proteins Rb, p130, and p107 [112–114]. Studies have shown that large T-antigen inhibits p53 activity by binding to its ATP domain [112]. LT recruits p130-E2F complexes via its LXCXE motif so that hsc70 bound to the J domain can use energy from ATP hydrolysis to free E2F that, in turn, binds to DNA to drive gene expression, S-phase entry, and cell proliferation [112, 114]. During viral-host cell protein interactions, five proteins named CUL7, CUL9, FBXW8, GLMN, and FAM111A are recruited to LT [120]. Some of these proteins act as cullin-RING ligases (CRLs) of an SCF (skp1, cullin, and F-box) type E3 ubiquitin ligase complex that targets cellular proteins for proteasomal degradation [121]. Recently, it was described that stable expression of LT protein in human fibroblasts is able to trigger the Rad3-related (ATR) kinase-DNA damage response (DDR) and that this leads to production of interferon and activation of the IFN regulatory factor 1 (IRF) transcription factor [122]. ATR promotes the expression of the p53 isoform Δp53, upregulation of the Cdk inhibitor p21, and, consequently, the downregulation of cyclin A-Cdk2/1 (AK) activity [123]. As a result, the host cells stay in the replicative S-phase, which is critical for viral genome amplification [123].

Recent studies have shown that overexpression of SV40 LT in mouse embryos fibroblasts (MEFs) leads to upregulation of transcription of many interferon stimulated genes (ISGs) including ISG56, OAS (2′-5′-oligoadenylate synthetase-1), Rsad2 (radical S-adenosyl methionine domain containing 2), Ifi27 (interferon alpha-inducible protein 27), and Mx1 (myxovirus resistance or interferon-induced GTP-binding protein), GTPases, P200 gene family, and PKR (protein kinase R), as well as IRF-7, IRF-9, RIG-1, STAT1, and STAT2 [124, 125]. STAT1 transcription factor was found to be a critical transducer of interferon stimulated genes and IFN-*β* in these SV40 expressing cell lines [122]. As expected, two SV40 gene regions, the J domain and adjacent LXCXE motif, were responsible for upregulation or downregulation of ISGs genes [125]. However, it is not known whether these biological events actually occur and what consequences would they have on thymus functionality in the immortomouse. We believe that MHC mediated antigen presentation and cell death processes that control positive and negative selection are normally occurring during thymopoiesis in the immortomouse. We assume that the expansion of CD4^+^ and CD8^+^ populations in the thymus could occur as a result of interferon-*α*/*β*-induced genes and enhanced survival of positively selected cells. The SV40 expressing T-cells may preserve the initial phase of activation-induced cell death (AICD) promoted by the Fas/CD95 proapoptotic pathway in response to self-RNA/DNA stimulation. It is well known that SV40 LT binds to p53 via core DNA-binding domain leading to reduced expression of apoptosis genes while increasing the expression of growth factor genes [112, 126, 127]. Currently, it is unclear whether and/or how Bim and Nur77 work together for induction of apoptosis and consequently negative selection [8, 128]. The cross talk between these signaling pathways and p53 in cell death needs to be further investigated.

FOXN1 gene expression is required for differentiation of the immature epithelial cells into functional cortical TECs (cTECs) and medullary TECs (mTECs). A recent study has shown that enforced expression of FOXN1 was capable of reprograming primary mouse embryonic fibroblasts (MEFs) into functional TECs [129]. Moreover, these induced TECs were able to promote full T-cell development* in vitro*. It seems that besides FOXN1 ectopic expression other stem cell transcription factors might play a significant role in the ensuing process. It is interesting that a subset of thymic epithelial cells express the transcription factors Nanog, Oct4, and Sox2 that are genes only expressed by totipotent stem cell [90]. To date, no study has investigated TEC cell lines derived from thymus of the tsA58 immortomouse which is under the control of the interferon inducible murine H-2K^b^ promoter. We supposed that expression of large T SV40 in adult TEC progenitors will increase mTECs and extended normal thymic regeneration and function, including the development of both CD4^+^ and CD8^+^ cells and the expression of MHC class I and II antigens. Nude mice are athymic and hairless because a mutation at FOXN1 gene blocks thymic epithelial cells patterning and differentiation in thymus and keratinocyte differentiation in the skin [98]. To investigate the possible roles of the embryonic thymic epithelial cells for T-cell growth and hyperplasia in immortomouse, we crossed a BALB/c immortomouse strain with Nude BALB/c mouse strain. Mice bearing FOXN1 deficiency and overexpression of SV40 Tag were healthy and developed normally and males were fertile. We did not observe thymus hyperplasia in the old mice. Further studies using these new transgenic mouse strains will be necessary for interrogating the role of cTEC and mTEC progenitors in the T-cell expansion and the mechanism of homeostatic lymphocyte trafficking and survival as well as the dynamic encounters of T-cells and dendritic cells during T-cell development inside the thymus.

Finally, thymic-related carcinomas (derived from thymic epithelial cells) are heterogeneous in types and difficult to diagnose [119, 120, 126]. Whereas tumor cells from thymomas look similar to the normal cells of the thymus and do not spread beyond the thymus, cells from thymic carcinoma grow more quickly than thymomas and usually spread to other parts of the body. Treatment protocols are not solidly established, and new drugs and treatments are awaiting discovery. The thymic hyperplasia observed in immortomouse is poorly understood [107]. The polyomaviruses may induce various types of cancers in animal models and humans, including B- and T-cell lymphomas [119, 120, 126]. Transgenic expression of c-terminal mutated forms of large T under the control of lymphotropic papovavirus promoter is able to induce brain tumors and thymic lymphomas in mice [130]. The cells lines derived from transgenic thymuses were homogeneous for one of the mature T-cell subsets, either CD4^+^ CD8^−^ or CD4^−^ CD8^+^ cells, which expressed the TcR/CD3 higher phenotype [130]. In another study, McCarthy and colleagues analyzed the role of SV40 T in apoptosis induced by p53 in positive and negative selection [131]. They concluded that T-antigen did not interfere with clonal deletion of thymocytes in transgenic mice suggesting normal development and that the autoreactive thymocytes were killed via p53-independent apoptosis [131]. In one model to explain mouse predisposition of T-cell lymphoma, it is proposed that T-antigen contributes to the survival of cells that have undergone DNA damage due to TCR gene rearrangement for positive and negative selection [131]. We do not know whether these mechanisms are similar in human tumors [119]. The tsA58 transgene is expressed in very low amounts under physiological condition in the immortomouse and only the thymus responds to its growth effects [107]. The protein stability is temperature-sensitive and stable at 33°C but not at 39°C, and experiments using many functionally immortalized cell lines did not turn in tumor formation when injected in Nude mice [107, 111]. Thus, the immortomouse is a model that suits well experimental research on induction of lymphomas and thymic carcinomas after crossbreeding with other transgenic or knockout mice bearing a candidate mutated oncogene or deleted tumor suppressor gene.

## 12. Conclusions And Perspectives

Naive CD4^+^ T-cell interactions with antigen-presenting DCs promote their commitment to specific effector lineages named Th1, Th2, Th9, and Th17. The follicular helper (Tfh) T-cells have emerged as a special T-cell subset defined by CXCR5 and Bcl-6 expression that drive the differentiation of cognate B-cells into memory and plasma cells, which are required for the generation of T-cell-dependent B-cell responses and antibodies production. The identification of Tfh cells provides promise for novel strategies to improve vaccine development and long-lived humoral protection against infectious disease.

Natural Treg from thymic origin and induced Treg cells from periphery lymphoid tissues as well as the Treg cell lineages Tr1 and Th3 can exert both beneficial and pathogenic effects. In certain settings, the choice between immunity versus tolerance depends on local or systemic responses to inflammatory and immunologic signals. CD8^+^ Tregs are induced after different immunotherapy regimen in various pathophysiological situations. There is a lot of exciting work to be done for a more comprehensive understanding of the cellular and biochemical mechanisms by which Treg cells exert their suppressive activities to control the relative balance of Th1/Th17 cells. To do so, we need to advance in biological methods to identify, isolate, and expand* ex vivo* generated Treg cell lines in mouse transplant models.

In the future, we need to develop pharmaceutical inhibitors and monoclonal antibodies to suppress inflammatory cytokines, such as TGF-*β*, IL-6, IL-9, IL-17, IL-22, and IL-23, dexamethasone, and vitamin D3 in clinical protocols to avoid redifferentiation and conversion of Treg cells into Th1/Th17 pathogenic phenotype. Such knowledge will contribute to the development of a more realistic strategy to avoid solid organ graft rejection and acquired tolerance leading to cancer progression or autoimmune diseases.

The immortomouse, or its immortalizing genetic approach, is one interesting mouse model for exploring the thymus organogenesis and T-cell development toward understanding the Treg biology and its tolerogenic potential for therapy in transplantation. The isolation and growth of the embryonic stem cell-derived thymic epithelial cells is one promising strategy to treat autoimmunity and thymus involution in patients. Thus, unique humanized mice models need to be designed and developed for investigating these new therapeutic concepts. Therefore, the generation of hybrid immortomice and humanized transgenic mice can enable the development of novel approaches that could significantly advance the development of T-cell subtypes and TECs-based therapies.
